# Influence of Root System Characteristics on Black Spruce Seedling Responses to Limiting Conditions

**DOI:** 10.3390/plants8030070

**Published:** 2019-03-19

**Authors:** Clémentine Pernot, Nelson Thiffault, Annie DesRochers

**Affiliations:** 1 Institut de recherche sur les forêts, Université du Québec en Abitibi-Témiscamingue, Amos, QC J9T 2L8, Canada; annie.desrochers@uqat.ca; 2 Canadian Wood Fibre Centre, Canadian Forest Service, Natural Resources Canada, Québec, QC G1V 4C7, Canada; nelson.thiffault@canada.ca

**Keywords:** initial roots, adventitious roots, stock type, black spruce, limiting resources, irrigation, fertilization, flooding

## Abstract

Roots directly affect planted seedling adaptation to new growing conditions at reforestation sites. To test the influence of root characteristics on the short-term response of seedlings to limiting resources (water, nutrient, or oxygen), we conducted two experiments. We compared (1) the growth and physiology of three types of four-year-old black spruce (*Picea mariana* (Mill.) BSP) seedlings (Containerized, highly developed initial roots restricted to a plug; bareroot, less developed but unrestricted initial roots; deeply-planted containerized, restricted initial and adventitious roots) to different combinations of irrigation and fertilization. We also investigated (2) the cellular plasticity of adventitious and initial roots to three irrigation regimes including flooding. Bareroot seedlings had better relative growth rates in height than containerized seedlings, probably due to their larger initial size. On the other hand, containerized seedlings took better advantage of fertilization, as shown by a higher relative growth rate in diameter compared to bareroot seedlings and were less affected by water limitation, possibly due to the root plug acting as an additional water reserve capacity. For containerized seedlings, the presence of adventitious roots was beneficial to height growth and physiological performances compared to seedlings with initial roots only. Adventitious roots showed great cell plasticity, particularly under flooding conditions.

## 1. Introduction

Black spruce (*Picea mariana* Mill. BSP) is a dominant species of North American boreal forests. It has a large ecological spectrum [1], able to grow in conditions ranging from peatlands [2,3] to mesic well-drained sites [4]. Its plasticity, as well as its fiber quality and resistance to pests and diseases, make it the most reforested species in Quebec, Canada [5]. With more than 75 million black spruce seedlings planted annually in this province, ensuring the success of plantations is fundamental to support sustainable forest management objectives.

The root system plays a key role in tree establishment [6]. This is especially important when outplanting seedlings from nursery to field conditions, because the range of changing conditions can be considerable. Seedling ability to develop new roots impacts the time required to establish direct contact with the planting site [7,8,9], which is necessary to have direct access to soil resources. Indeed, water stress is the main cause of conifer mortality after planting [10,11], and limited access to nutrients may cause post-planting growth stagnation [10,12]. During nursery production, the ability to produce new roots is thus an important criterion for selecting the most vigorous seedlings [13,14,15].

Two stock types are commonly used in plantations: (1) Seedlings produced in rigid-walled containers, resulting in highly developed root systems restricted in a root plug; and (2) bareroot seedlings grown in open fields, producing unrestricted root systems but of a lower biomass [16,17]. These different root traits can influence seedling aboveground development and their physiology. When reforestation sites present potentially harsh growing conditions, containerized seedlings are often favored since the root plug can act as a source of water and nutrients for some time after planting, allowing them to have greater root growth potential and to be more resistant to water stress than bareroot seedlings [18,19,20]. On the other hand, bareroot stock is generally preferred for outplanting on sites subjected to high levels of competition as bareroot seedlings are usually larger than container-grown stock [21].

Containerized and bareroot seedlings however, present root structures that differ from those of naturally regenerated seedlings. Indeed, black spruce planting stock are produced and planted at the root collar level, resulting in root systems exclusively composed of initial roots, i.e., roots formed below the seed germination point. In contrast, the root system of black spruce is predominantly formed from adventitious roots under natural conditions [4,22,23]. Adventitious roots develop at the base of the stem for the first years and gradually replace initial roots. In many species, the formation of adventitious roots may allow a better adaptation to harsh environmental conditions, such as flooding [24,25]. This is the case for tamarack (*Larix laricina* (Du Roi) K. Koch), which shares the dominance of boreal peatlands with black spruce [2,26]. The adventitious roots of this species have different cellular morphologies, with less developed endodermis and a delayed suberization compared to initial roots, allowing tamarack seedlings to increase apoplastic water transport and thus maintain water balance despite anaerobic conditions [27]. Adventitious roots can be formed with other cellular structures to adapt to flooding such as aerenchyma—tissues with large intercellular air-filled spaces that facilitate oxygen diffusion [28,29]. In some herbaceous species, adventitious roots can also facilitate nutrient uptake. For example, P uptake rate of *Solanum dulcamara* L. was dependent on adventitious root biomass [30], while *Triticum aestivum* L. exhibited increased P and K uptake with adventitious nodal roots compared to seminal roots under stagnant flooded conditions [31]. We did not observe significant differences in the ability of black spruce seedlings to uptake nutrients from adventitious or initial roots separated, under greenhouse-controlled conditions [32]. However, trees with adventitious and initial roots had increased soil nutrients uptake (particularly nitrogen) compared to seedlings with only initial roots under field conditions [33].

**Figure 1:**
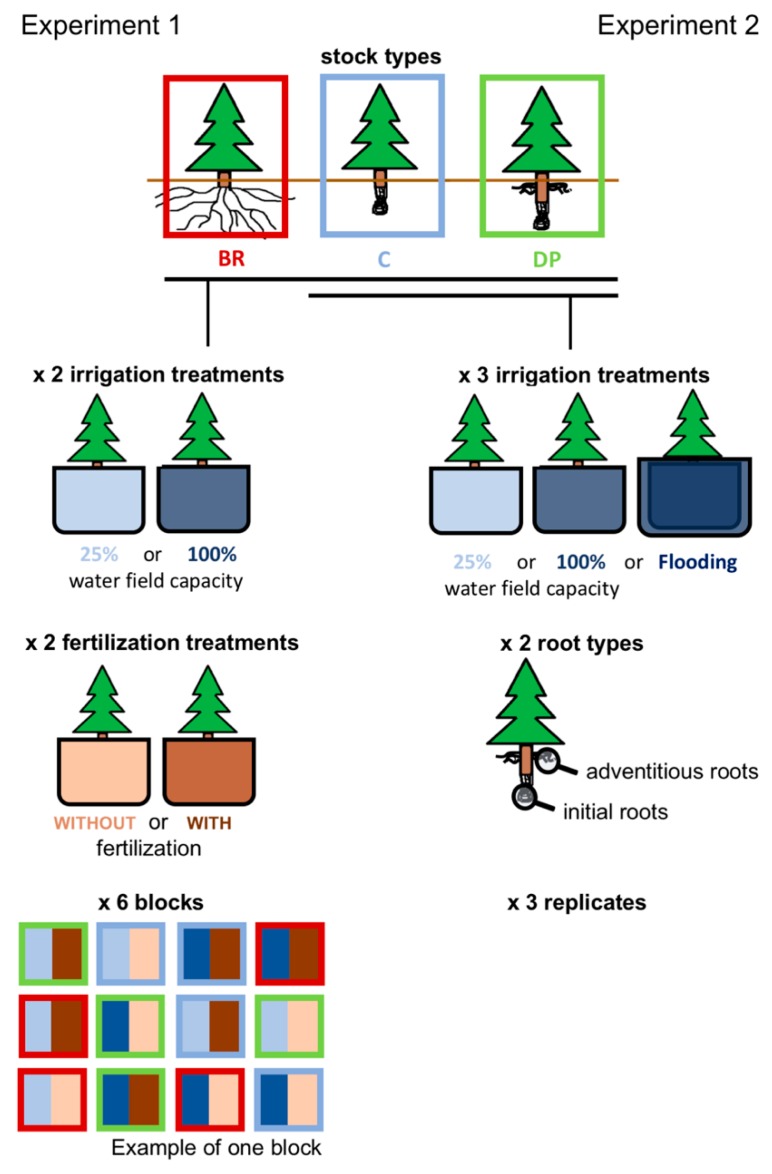
Greenhouse experiments using four-year-old black spruce seedlings. Experiment 1 consisted of three stock types: BR, bareroot seedlings (with an unrestricted root system, mainly composed of initial roots); C, containerized seedlings (with a restricted initial root system); and DP, deeply-planted containerized seedlings (with initial and adventitious roots restricted in a root plug). These were subjected to two irrigation treatments (maintenance of 25% or 100% water field capacity) and two fertilization treatments (with or without fertilization), replicated six times in a factorial design. Experiment 2 consisted of C and DP seedlings subjected to three irrigation treatments (maintenance of 25% or 100% water field capacity, or flooding condition). Cell morphology was observed for initial and adventitious roots (initial roots only for C seedlings) on three replicates per treatment.

In this study, we hypothesized that black spruce seedlings with adventitious roots adapt more easily to limiting conditions (water, nutrient, or oxygen) than seedlings with initial roots only, by having (1) better water and nutrient uptake under limited resources availability and (2) greater cellular plasticity that facilitates adaptation to different irrigation conditions. We tested our hypothesis using two experiments. In Experiment 1, we first tested water and nutrient uptake of three stock types that had previously shown differential responses when outplanted on a boreal site [33]: (C) Containerized seedlings, the most commonly used stock type in potentially harsh planting conditions, with initial roots restricted in a root plug; (DP) deeply-planted containerized seedlings, also produced in a container but with the root collar buried 5 cm to develop adventitious roots in addition to the initial roots; and (BR) bareroot seedlings, with a root system developed without space restriction but composed mainly of initial roots. We subjected seedlings to two irrigation treatments (25% or 100% water field capacity) and two levels of fertilization (with or without fertilizer), and also compared growth and physiology among stock types (Figure 1). Secondly, knowing that adventitious roots develop both under high water-content [34] and well-drained [4] conditions, we conducted Experiment 2 by comparing the cellular morphology of adventitious and initial roots under different irrigation regimes: (i) Limited water supply (25% water field capacity), (ii) well-watered conditions (100% water field capacity), and (iii) flooding conditions (Figure 1). Since adventitious roots are often an adaptive response [35,36], we anticipated that DP seedlings would be more suitable for planting on sites with harsh growing conditions, such as those submitted to flooding or water and nutrient limitations.

## 2. Results

### 2.1. Experiment 1: Growth and Physiological Responses to Irrigation and Fertilization Treatments

**Figure 2:**
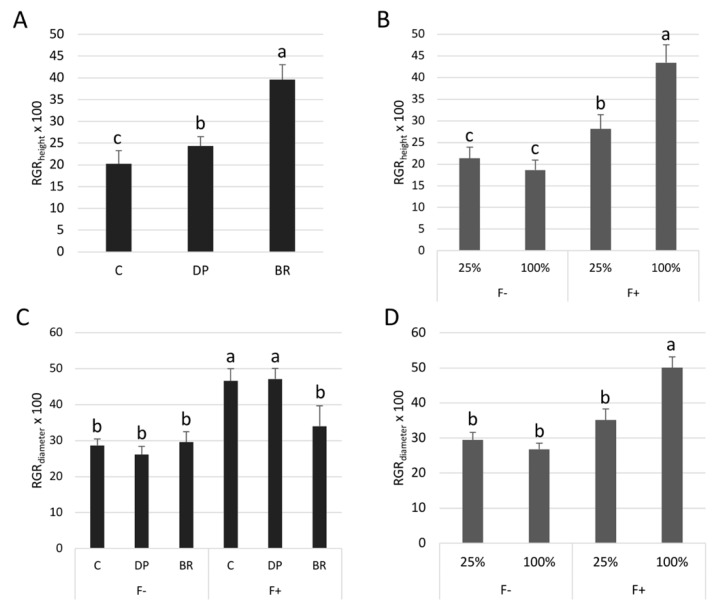
Relative growth rate in height (RGR_height_) of black spruce seedlings according to (**A**) stock types and (**B**) irrigation (25% or 100% field capacity) × fertilization (F− without and F+ with fertilization) over one growing season. Relative growth rate in diameter (RGR_diameter_) of seedlings according to (**C**) stock type × fertilization and (**D**) irrigation × fertilization over one growing season. Bars with the same letter indicate a non-significant difference at *P* ≤ 0.05.

Bareroot (BR) seedlings had the greatest relative growth rate in height (RGR_height_) after 14 weeks (representing a gain of about 40% of initial height), twice the height growth of containerized (C) seedlings. Deeply-planted containerized (DP) seedlings had intermediate RGR_height_ with an average increase of 24% (Figure 2A). RGR_height_ was also influenced by the interaction between fertilization and irrigation treatments (Table 1). Unfertilized seedlings had low and similar RGR_height_ under both irrigation treatments while irrigation increased the height growth of fertilized seedlings (Figure 2B). Fertilization also increased relative growth rate in the basal diameter (RGR_diameter_) of C and DP seedlings, while it did not increase diameter growth of BR seedlings, independently of the irrigation treatment (Table 1, Figure 2C). The high irrigation level increased the RGR_diameter_ of fertilized seedlings only (Figure 2D).

**Figure 3:**
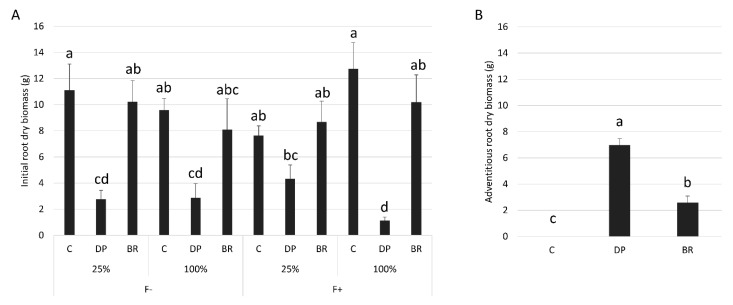
Mean dry biomass of black spruce (**A**) initial roots for each stock type × irrigation (25% or 100% field capacity) × fertilization (F− without and F+ with fertilization) treatments and (**B**) adventitious roots for each stock type. Bars with the same letter indicate a non-significant difference at *P* ≤ 0.05.

The total biomass of BR seedlings at the end of the experiment was greater than that of C and DP seedlings (Table 2). Fertilization increased the total biomass of seedlings while irrigation had no significant effect (Table 1 and Table 2). Total root biomass was similar for all treatment combinations (Table 1 and Table 2). However, the proportions of initial vs. adventitious roots differed between the treatments. Root systems of C and BR seedlings were predominantly composed of initial roots, with greater initial root biomass than DP seedlings, except for fertilized seedlings under limited irrigation where differences were not significant (Figure 3A). Conversely, DP seedlings developed more adventitious roots than C and BR seedlings, however, BR seedlings developed a small number of adventitious roots while C seedlings exclusively had initial roots (Figure 3B). Seedlings had similar final root/shoot ratios among stock types and water regimes (Table 1), but unfertilized seedlings invested more in root development than in aerial parts compared to fertilized ones (Table 2).

**Figure 4:**
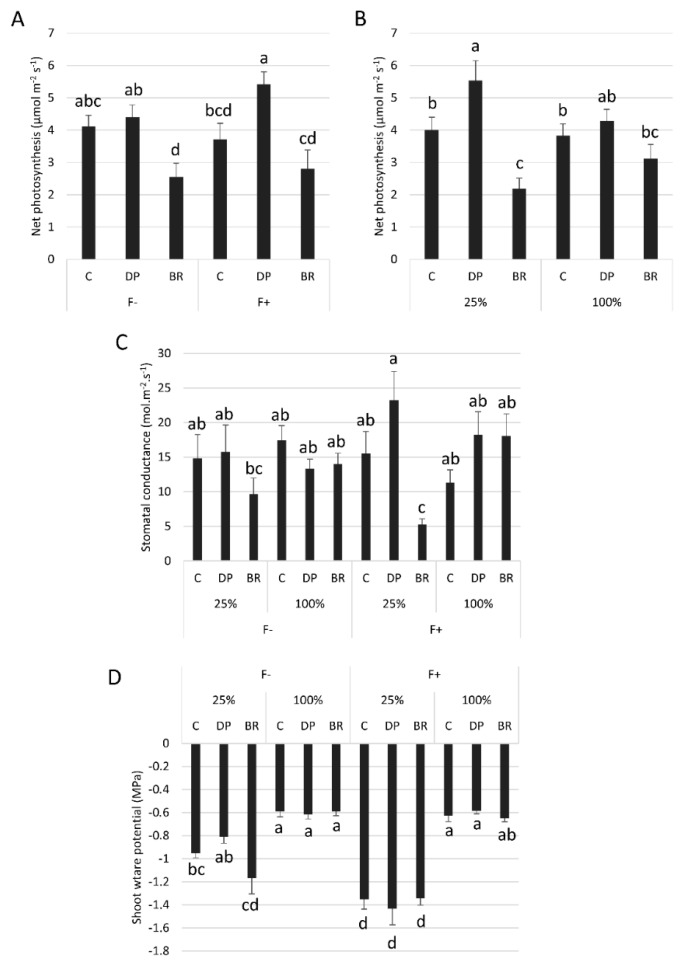
Mean net photosynthesis according to (**A**) stock type × fertilization (F− without and F+ with fertilization) and (**B**) stock type × irrigation (25% or 100% field capacity). Effects of stock type × irrigation × fertilization on (**C**) stomatal conductance and (**D**) shoot water potential. Bars with the same letter indicate a non-significant difference at *P* ≤ 0.05.

The net photosynthesis response of seedlings to fertilization and irrigation differed among stock types (Table 1). Unfertilized C and DP seedlings had a higher net photosynthesis than BRs, whereas fertilized DP seedlings had higher net photosynthesis than the other two stock types (Figure 4A). Well irrigated seedlings of all stock types had similar mean net photosynthesis, while DP seedlings had greater net photosynthesis than C and BR seedlings under water-limited conditions (Figure 4B). Similarly, stomatal conductance of BR seedlings was also more reduced by water limitation compared to C and DP seedlings, especially in the presence of fertilization, since this combination of treatments was the only one to have resulted in a significantly greater stomatal conductance in DP seedlings compared to BRs (Figure 4C). As expected, shoot water potential was lowest under limited irrigation conditions, especially for fertilized seedlings (Figure 4D). Stock type or fertilization had no effect on shoot water potential under well-watered conditions. Under water limiting conditions, fertilization significantly reduced the shoot water potential of all stock types compared to the well-watered condition, while without fertilization only C and BR seedlings had low shoot water potential, DP seedling shoot water potential was similar to that of well-watered seedlings (Figure 4D).

**Figure 5:**
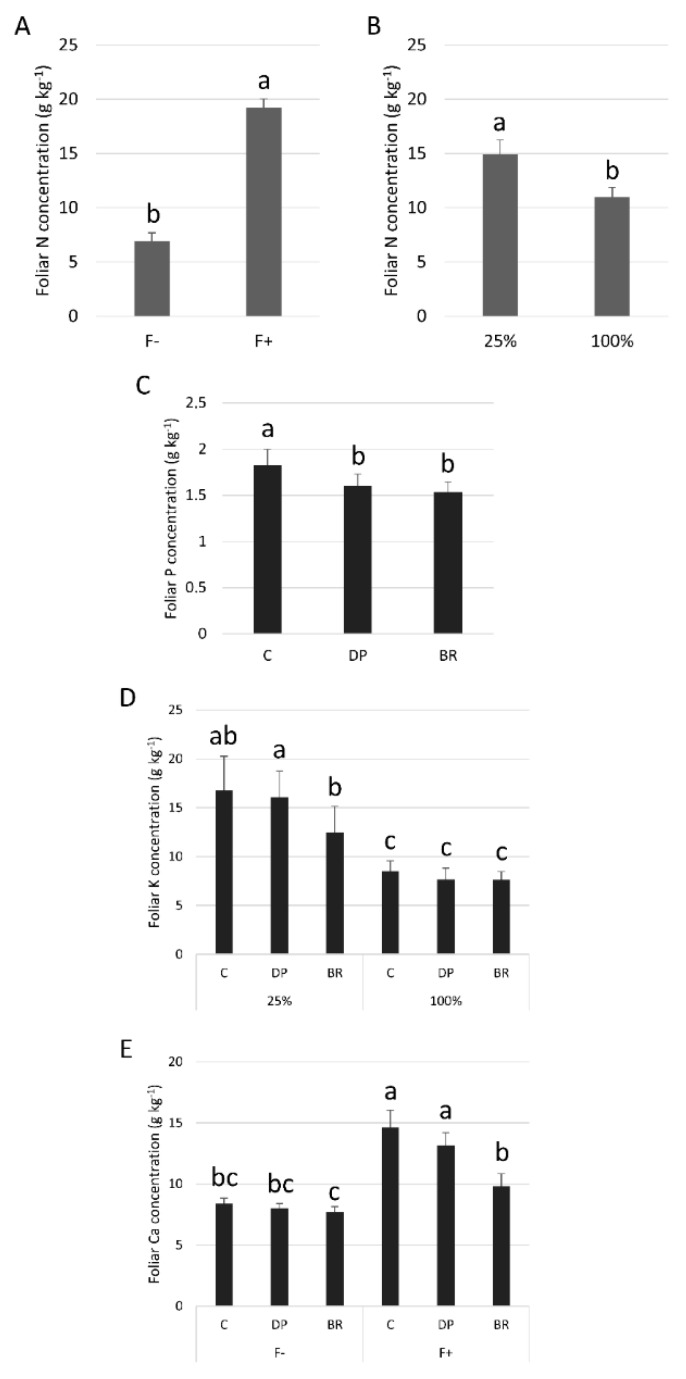
Mean foliar nutrient concentrations of black spruce seedlings: N, nitrogen according to (**A**) fertilization (F- without and F+ with fertilization); (**B**) irrigation (25% or 100% field capacity); (**C**) P, phosphorus according stock type; (**D**) K, potassium according stock type × irrigation; and (**E**) Ca, calcium according to stock type × fertilization. Bars with the same letter indicate a non-significant difference at *P* ≤ 0.05.

Needle N concentrations were similar for the three stock types (Table 1). Fertilization increased N concentrations in needles (Figure 5A), while irrigation decreased it (Figure 5B). C seedlings had greater P concentrations in needles than DP and BR seedlings (Figure 5C). There was an interaction between irrigation and fertilization treatments such that irrigation had no effect on needle P concentrations of fertilized seedlings while unfertilized seedlings had greater P concentrations under the low irrigation treatment (Figure S1A). Droughted DP seedlings had greater K concentrations than BR seedlings (16.1 ± 2.5 g kg^−1^ vs. 12.4 ± 2.6 g kg^−1^; mean ± SE), while C seedlings, having a larger response variability, had an intermediate status (16.7 ± 3.6 g kg^−1^) (Figure 5D). Fertilized C and DP seedlings had higher Ca concentrations compared to BR seedlings, while this trend was not significant for unfertilized seedlings (Figure 5E). Finally, fertilized seedlings had greater K and Ca concentrations under low irrigation, while unfertilized seedlings had low and similar concentrations (Figure S1B,C).

### 2.2. Experiment 2: Root Cell Morphology

**Figure 6:**
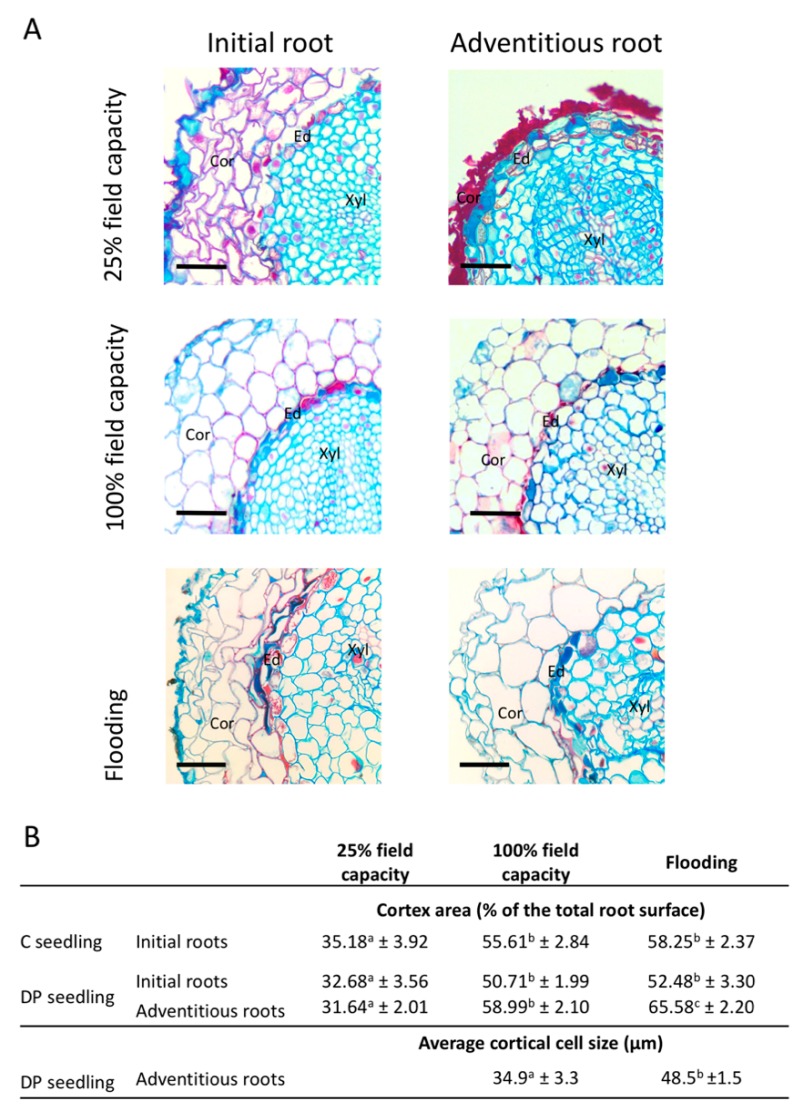
Initial and adventitious root morphology of black spruce seedlings under different irrigation regimes. (**A**) Cross-section of initial and adventitious roots of seedlings irrigated at 25% field capacity, 100% field capacity, or flooded. Sections were taken at 20–40 mm from the root tip (Bars = 50 μm; Ed, Endodermis; Cor, Cortex; Xyl, Xylem). (**B**) Significant measurements of cortex area and average cortical cell size of initial and adventitious roots of seedlings irrigated at 25% field capacity, 100% field capacity, or flooded, according their stock types. Values are grand median ± SE. Different letters in the same row indicate significant differences at *P* ≤ 0.05.

At 25% water field capacity (FC), seedlings produced initial and adventitious roots with a smaller cortex than seedlings at 100% FC or under flooding conditions (Figure 6). Seedlings irrigated at 25% FC exhibited adventitious roots composed of cortical cells with suberized cell walls but that were completely collapsed, forming a thick suberized layer on the periphery of the endodermis. Collapsed suberized cortical cells were also observed in initial roots under limited water supply, but in a less systematic way (Figure S2). In the case where cortical cells were not collapsed, they still showed suberized cell walls (Figure 6A). For both initial and adventitious roots of seedlings under limited water regime, endodermis appeared more suberized than in roots of seedlings under 100% FC or flooded. At 100% FC, the cell morphology of initial and adventitious roots was very similar (Figure 6A). Flooding induced different responses in initial and adventitious roots, adventitious roots had larger cortical cells forming a bigger cortex area than those of seedlings under the other water regimes, whereas the initial roots of flooded seedlings had similar cortex areas to seedlings under 100% FC (Figure 6). The appearance and size of the root cortex were similar in initial roots developed by C or DP seedlings under all irrigation treatments (Figure 6B).

## 3. Discussion

After one growing season, nutrient limitation had more impact on seedling growth than water restriction (Table 1, Figure 2B) and nutrient uptake exacerbated water stress (Figure 2B,D and Figure 4D), as was found elsewhere [37,38]. Black spruce is known to respond well to fertilization in terms of growth and nutrient uptake [18,39,40], confirmed here by positive responses in RGR_height_, biomass, and nutrient foliar concentrations in fertilized seedlings (Figure 2 and Figure 5, Figure S1). Unlike what was observed elsewhere [41,42], fertilization did not increase the net photosynthesis of the different seedling stock types. Some *Pinus* species as well as *Pseudotsuga menziesii* have also been shown to maintain stable photosynthesis rates despite nutrient additions [43,44,45,46]. Warren et al. [46] proposed that in these cases, the increase in leaf area and biomass allocation changes are the main responses to fertilization. In our study, fertilized seedlings indeed invested more in aboveground biomass production than those without fertilization (Table 2), as reported elsewhere [26,47]. The relatively low impact of irrigation may reflect that our water limitation treatment did not create sufficiently stressful conditions to reduce growth or biomass, especially without fertilization. Indeed, although 25% FC was enough to decrease growth and survival of one-year-old black spruce seedlings [34], here it resulted in average shoot water potentials of −1.43 and −1.17 MPa at the lowest—with or without fertilization, respectively (Figure 4D)—whereas the osmotic potential at turgor-loss point of black spruce is between −1.44 and −2.36 MPa [48,49]. Nevertheless, based on a water stress criterion defined by a water potential of about −1.10 MPa, Patterson et al. [26] found that one-year-old seedling growth was more affected by irrigation than by fertilization. This may indicate that after a period during which black spruce seedling growth would be highly dependent on water uptake immediately after germination [26], the trend is reversed in subsequent years with a greater need for nutrients. This seems to be in line with observations we have made in the field, where four-year-old black spruce seedlings had better growth on the richest but driest microsites two years after planting [33].

Bareroot (BR) seedlings had better aboveground growth (Figure 2A, Table 2) but were more physiologically affected by water restriction compared to containerized (C) and deeply-planted containerized (DP) seedlings (Figure 4B–D). At the beginning of the experiment, BR seedlings were slightly larger in diameter than C and DP seedlings (significantly different at *P* ≤ 0.06). Although no biomass measurements were made at the onset of the experiment, we can assume that BR seedlings had a greater biomass (at least aboveground) than the other stock types [50,51], which could have influenced final biomass values. Larger initial diameters are associated with greater height growth, even when assessed over more than one growing season [52]. Indeed, bigger seedlings form buds containing a larger number of needle primordia, which drive predetermined shoot growth (unaffected by the environmental conditions) during the next growing season [53,54]. Differences in initial seedling size might thus explain the overall greater height growth of BRs relative to other stock types [17], regardless of resource availability (Figure 2A).

In addition to a generally more developed root system than BRs at the time of planting [19,55,56], containerized seedlings have the advantage of having a root plug, which acts as a source of moisture and nutrients during and just after planting [18,20,57]. For example, Norway spruce (*Picea abies* L. Karst.) seedlings, with root plugs wet to saturation before planting, had much reduced mortality rates until three to four weeks of drought compared to dry root plug seedlings [58,59]. In our study, even if submitted to a moderate water stress, BR seedlings were more rapidly impacted by limited irrigation compared to containerized seedlings. We observed a similar trend in response to increased nutrient availability, with containerized seedlings exhibiting higher foliar nutrient concentrations than BRs (although not for N, the most limiting nutrient for the development of black spruce under natural conditions (Figure 5) [60,61]). Since the root system of BR seedlings is trimmed when trees are lifted at the nursery before outplanting in the field, initial root/shoot ratios are most likely unbalanced at planting compared to the other stock types [56,62], which could explain their greater sensitivity to water limitations [63].

Containerized seedlings had a greater RGR_diameter_ than BR seedlings in the presence of fertilization, independently of the water regime (Figure 2C). High density imposed by containerized seedling production can induce slimmer seedlings due to restricted light access [64,65], as diameter growth is sensitive to light levels [66,67]. Once transferred from containers to large pots, C and DP seedlings probably had better light access and invested more in diameter growth, just as trees reaching the canopy submitted to lower light competition are able to invest more in diameter growth than in height under field conditions [68,69]. For BRs that are produced in open fields at a generally lower density than containerized seedlings, change of light intensity may have been less critical. However, under limiting nutrient and water conditions, all stock types expressed the same RGR_diameter_ (Figure 2). Diameter growth is more affected by environmental conditions of the current growing season than height growth [54,70]. So, as containerized seedlings are known to perform better than BR seedlings in harsh environments [17,18], and in view of the results we obtained using the same stock types planted in boreal microsites [33], it appears that our semi-controlled growth conditions were probably only moderately stressful. Limiting irrigation treatment resulted in moderate water stress and, although the nutrient limitation was restrictive, black spruce is a species usually growing in nutrient limited environments [61,71].

The presence of adventitious roots in containerized seedlings has been beneficial to growth, with higher RGR_height_ of DP compared to C seedlings (Figure 2A). This could be explained by slightly improved physiological performances; seedlings with adventitious roots had better net photosynthesis and shoot water potential than the other seedling types under water-limited conditions (Figure 4A,B,D). However, this did not translate into greater RGR_diameter_ within the short time frame of our experiment (Figure 2C). The higher RGR_height_ of DP seedlings could also be explained by a better morphological adaptation of adventitious roots to their environment compared to the initial roots. First, when submitted to a moderate water stress, both initial and adventitious roots underwent cellular morphological changes (Figure 6). Root cortex death may be one of the root responses to water stress [72,73,74]. Although strongly reducing hydraulic root conductance and ion uptake, cortex death associated with suberized endodermis protects the stele from drought and therefore the vascular tissues, thus allowing plants to maintain water transport within the root system [72,74,75,76]. Once more favorable water conditions have been restored, bare stele roots are able to resume their elongation and regenerate new lateral roots [72,73,77]. For black spruce seedlings, protection of the stele by suberized endodermis appears to have been enhanced by the maintenance of collapsed cortical cells forming a thick suberin barrier on the endodermis periphery (Figure 6). This phenomenon has been observed in all adventitious roots and in about two-thirds of initial roots (Figure S2). This may explain why unfertilized DP seedlings under the low irrigation treatment could maintain similar water potentials to well-irrigated seedlings (Figure 4D). The ability of adventitious roots to change their cell morphology was particularly visible under flooding conditions (Figure 6), with adventitious root formation being a response of flood-tolerant species [24,25]. Root morphological adaptations to flooding occur mainly in the cortex area, usually as aerenchyma [78,79], but also in the form of larger cortical cells generally associated with larger intercellular spaces, allowing roots to have better porosity [80,81]. These adaptations usually occur within a few days or weeks but may require long-term stressful conditions to affect physiology and seedling growth [30]. Flooding conditions create an oxygen gradient in the soil that promotes root growth close to the soil surface where oxygen availability is highest [24,82]. In our study, only adventitious roots were able to adapt quickly to flooding, and this would not only be promoted by an advantageous location close to the soil surface, since initial roots of C seedlings that were also located near the soil surface were not able to adapt in the short term.

In conclusion, the presence of adventitious roots was beneficial for containerized black spruce seedlings, resulting in improved physiological performance and greater height growth in DP seedlings relative to C seedlings. Our results show that the physiological advantage provided to seedlings by adventitious roots occurred mainly under limited water conditions and could be related to the greater cellular plasticity of adventitious vs. initial roots. This better cell plasticity was particularly observed in flooding condition. Thus, seedlings with adventitious roots could perform better in planting sites subjected to drought or flooding events, compared to seedlings with initial roots only. On the other hand, BR seedlings had a better relative growth rate in height than containerized seedlings, probably due to a bigger initial size, giving them a short-term advantage in sites with moderately stressful conditions.

## 4. Materials and Methods

Three stock types of 4-year-old black spruce seedlings were used for the experiments: (C) Containerized seedlings produced in 110 cm^3^ cavities, planted at the root collar level (initial root system restricted in a root plug); (DP) deeply-planted containerized seedlings, produced in 110 cm^3^ cavities with the root collar buried 5 cm belowground at 1-year-old (initial and adventitious root systems restricted in a root plug); and (BR) bareroot seedlings, produced in outdoor growing beds (unrestricted initial root system) (Figure 1). C and DP seedlings originated from local seed sources 49°49′ N, 74°45′ W and BRs from 48°12′ N, 71°29′ W. One year before the experiments started, seedlings of each stock type were transferred in 10 dm^3^ containers for Experiment 1 and 4 dm^3^ containers for Experiment 2. Planting cavities were filled with a peat, perlite, coconut husk fiber substrate (Fafard, AGRO MIX^®^ PV20). For both experiments, seedlings were grown in a semi-controlled greenhouse environment. Natural light was supplemented with 400 W high pressure sodium S51 lamps (Kavita Canada Inc., Niagara-on-the-Lake, ON, Canada) that provided a photosynthetic flux density of about 400 µmol photon m^−2^ s^−1^ at leaf level; light/dark photoperiod was set to 16 h/8 h, associated with temperatures of 25 °C/17 °C, respectively; natural humidity levels were maintained.

### 4.1. Experiment 1

In February 2013 and for 14 weeks, seedlings were subjected to a factorial irrigation × fertilization combination of treatments—100% or 25% FC and with (a weekly addition 0.2 g of NPK soluble mineral fertilizer [20-20-20]) or without fertilization. The experiment was realized as a randomized factorial design with six replicate blocks, each block being composed of the twelve combinations of stock type (BR, C, or DP) × irrigation (100% or 25% FC) × fertilization (with or without fertilizer) (Figure 1). To avoid confounding the fertilizer treatment with the irrigation treatment, volumes and concentrations of fertilizer were adjusted to 200 mL of 1 g L^−1^ of NPK fertilizer for 100% FC treatment or 100 mL of 2 g L^−1^ of NPK fertilizer for 25% FC treatment. Soil water content was monitored daily with a moisture probe (TDR 100; FieldScout, Portland, OR, USA) and tap water was added as needed.

Physiological measurements were taken at the end of the 14-week experiment. Net photosynthesis and stomatal conductance were measured between 10:00 and 15:00 using a LI-6400 portable infra-red gas analyzer (LI-COR, Lincoln, NE, USA) equipped with a conifer cuvette (CO_2_ concentration of 400 µmol mol^−1^; leaf temperature of 24 ± 1 °C; 1000 µmol photons m^−2^ s^−1^ with a halogen lamp placed above the cuvette) using 1-year-old twigs from the seedlings’ upper-half. Leaf surface area of the measured twigs was determined using a LI-3100 Leaf Area Meter (LI-COR, Lincoln, NE, USA), and gas exchange values were calculated accordingly. Shoot water potential was measured at the end of the experiment between 7:00 and 10:00 using a Scholander pressure chamber (Model 1000; PMS Instruments, Albany, OR, USA) on 3 shoots per seedling, located in the upper-half section of the stem.

Seedling height (cm) and basal diameter (mm) were measured at the beginning and end of the experiment. Initial height and diameter were respectively (mean ± SD): 33.5 ± 4.5 cm and 7.1 ± 0.8 mm for C seedlings, 34.1 ± 5.1 cm and 6.9 ± 1 mm for DP seedlings, and 31.4 ± 4 cm and 7.8 ± 1.2 mm for BR seedlings. Relative growth rates (RGR) in height and basal diameter were estimated using the difference of the natural logarithm of the final and initial measurement of the growing season. After the 14 weeks, seedlings were divided into subparts to determine dry biomass—stems, needles, adventitious roots, and initial roots were oven dried for 48 h at 60 ± 5 °C. Dried needles were ground using a ball mill (Pulverisette 0, Fritsch, Idar-Oberstein, Germany) in order to perform nutrient analyses. Samples were analyzed with a TruMac N (LECO Corp., St Joseph, MI, USA) for N concentrations or with a plasma atomic emission spectrometer (Thermo Jarrel-Ash-ICAP 61E, Thermo Fisher Scientific, Waltham, MA, USA) for P, K, and Ca concentrations.

### 4.2. Experiment 2

In March 2013, C and DP seedlings were subjected to three types of irrigation. (1) Maintenance of 25% water field capacity, (2) 100% water field capacity, or (3) flooding—where containers were placed in bigger waterproof containers and covered with water. Three replicates of irrigation × stock type (C or DP) treatments were done (Figure 1).

Roots were examined after 14 weeks of growth under these conditions. Sampling was realized on C seedlings that only had initial roots and on DP seedlings that had initial and adventitious roots. Roots (5 cm long from the tip, outside the root plug) of each seedling were cut with a razor blade and rapidly placed on ice in PFA (paraformaldehyde 4% (*w*/*v*) in phosphate buffered saline (PBS, pH 7)). To ensure good fixation, several cycles of vacuum infiltration were achieved in a vacuum chamber before being incubated overnight at 4 °C. For tissue fixation and embedding, the protocol was adapted from Brewer et al. [83] with progressive gradients 20–40–60–80–95–100% of ethanol in PBS (each step for 1 h at room temperature, the 95% step overnight), 25–50–75–100% of xylene in ethanol at room temperature (each step during 1 to 2 h), then 50–100% of xylene in paraplast at 56 °C (50% for 4 h, 100% overnight).

Serial 7 μm sections were done with a Leica SM2400 sliding microtome (Leica Instruments GmbH, Hubloch, Germany). Sections were dewaxed using two 15-min baths of xylene and rehydrated by gradient 5-min baths of ethanol to water (100–100–95–70–50–0–0%). Double staining was performed to increase cell contrast—10 min of 1% aqueous safranin (red staining of lignified tissues) followed by 20 min of 1% aqueous alcian blue (bleu staining of cellulosic tissues). Sections were observed with a ZEISS Axioscope microscope (Carl Zeiss, Don Mills, Ontario, Canada). Measurements and analyses were performed with Fiji software [84]. All sections were measured to define root surface distribution (cortex area, *n* ≥ 18) and cortical cell sizes on a minimum of 6 sections of different roots per treatment.

### 4.3. Statistical Analyses

Analyses were performed in R v3.2.3 environment [85]. The influence of stock type, irrigation and fertilization on seedling growth and physiology was analyzed using a linear mixed effects model, using the function *lme* of the *nlme* library [86]. Response variables were RGR in height and diameter, dry biomass, net photosynthesis, stomatal conductance, shoot water potential, and foliar nutrient contents. The tested fixed effects were stock types, irrigation, and fertilization treatments and their interactions. Blocks were added to the model as a random effect. Data transformations were used when necessary to respect normality and homoscedasticity assumptions. Relative growth rate in height, initial and total root biomass, stomatal conductance, and foliar nutrient concentrations were *log*-transformed, while adventitious root biomass was *log*(*x* + 1)-transformed due to the occurrence of many zeros. When fixed effects or their interactions were significant, Tukey’s HSD post-hoc tests were performed using the *lsmeans* function of the *lsmeans* package [87]. When we detected a significant interaction between treatments for a given variable, we focused result presentation and our interpretations on the combination of treatments rather than on the individual effects of the treatments involved in the interaction. Influence of irrigation treatment was tested on the cortex area and average cortical cell size of each stock and root type separately, using the *aov* function followed by a Tukey’s HSD post-hoc test if the treatment was significant. *P* ≤ 0.05 was used as significance level in all analyses.

## Figures and Tables

**Table 1:** Effects of fertilization, irrigation, and stock type on growth, physiology, and nutrient concentration of four-year-old black spruce seedlings. When required, analyses were performed on transformed data (indicated in parentheses). Statistically significant values at *P* ≤ 0.05 are in bold.

Variables	Fertilization		Irrigation		Stock Type		Fertilization × Irrigation		Fertilization × Stock Type		Irrigation × Stock Type		Fertilization × Irrigation × Stock Type	
	*F*-Value	*P*-Value	*F*-Value	*P*-Value	*F*-Value	*P*-Value	*F*-value	*P*-Value	*F*-Value	*P*-Value	*F*-Value	*P*-Value	*F*-Value	*P*-Value
***Growth***														
RGR_height_ *(log)*	43.88	**<0.001**	3.68	0.060	25.89	**<0.001**	8.64	**0.005**	0.94	0.396	1.36	0.266	0.52	0.595
RGR_diameter_	37.52	**<0.001**	6.30	**0.015**	2.14	0.127	13.85	**0.001**	4.88	**0.011**	1.34	0.271	0.09	0.912
Total dry biomass	21.54	**<0.001**	0.49	0.486	5.21	**0.009**	0.79	0.379	0.59	0.560	2.07	0.137	1.67	0.198
Shoot dry biomass	45.52	**<0.001**	0.58	0.449	5.92	**0.005**	0.18	0.669	1.37	0.263	1.62	0.207	0.71	0.495
Total root dry biomass *(log)*	0.04	0.852	0.20	0.658	2.26	0.115	1.46	0.232	0.32	0.727	2.31	0.109	2.96	0.060
Adventitious root dry biomass *(logx* + 1*)*	0.01	0.993	0.80	0.376	103.28	**<0.001**	0.01	0.907	0.68	0.509	1.03	0.363	0.03	0.971
Initial root dry biomass *(log)*	0.31	0.581	2.39	0.128	47.84	**<0.001**	0.04	0.845	0.37	0.691	3.49	**0.038**	4.32	**0.018**
Root/Shoot ratio	35.80	**<0.001**	0.01	0.937	0.57	0.567	0.82	0.369	1.24	0.298	0.20	0.815	2.33	0.107
***Physiology***														
Shoot water potential	22.18	**<0.001**	174.92	**<0.001**	1.20	0.308	20.61	**<0.001**	1.35	0.269	0.78	0.464	3.23	**0.047**
Net photosynthesis	0.99	0.324	0.47	0.497	15.96	**<0.001**	1.65	0.203	4.28	**0.043**	3.62	**0.034**	2.62	0.082
Stomatal conductance *(log)*	0.05	0.833	4.75	**0.034**	7.25	**0.002**	0.01	0.944	2.96	**0.060**	9.87	**0.000**	3.89	**0.027**
***Nutrient concentration***														
N *(log)*	1092.67	**<0.001**	101.54	**<0.001**	0.28	0.755	0.38	0.542	2.60	0.064	1.32	0.279	0.41	0.668
P *(log)*	313.57	**<0.001**	3.40	0.071	3.15	**0.050**	4.02	**0.048**	1.73	0.187	1.29	0.285	2.82	0.069
K *(log)*	353.91	**<0.001**	77.33	**<0.001**	2.18	0.123	36.82	**<0.001**	2.37	0.104	3.91	**0.044**	1.29	0.285
Ca *(log)*	85.11	**<0.001**	29.90	**<0.001**	9.80	**0.000**	11.40	**0.001**	3.69	**0.032**	1.19	0.312	1.70	0.194

RGR_height_ = Relative growth rate in height; RGR_diameter_ = Relative growth rate in basal diameter.

**Table 2:** Mean (± SE) of shoot, root, and total dry biomass and root/shoot ratios according to fertilization (F− without and F+ with fertilization) and stock type (C, containerized seedlings; DP, deeply-planted containerized seedlings; and BR, bareroot seedlings (refer to Figure 1 for description)). Different letters for the same fixed effect (fertilization or stock type) indicate significant differences at *P* ≤ 0.05.

Variables	Fertilization		Stock Type		
	F−	F+	C	DP	BR
Shoot dry biomass (g)	18.4 ^b^ ± 0.8	27.1 ^a^ ± 1.1	20.6 ^b^ ± 1.1	21.9 ^b^ ± 1.4	25.6 ^a^ ± 1.8
Root dry biomass (g)	10.6 ^a^ ± 0.6	10.7 ^a^ ± 0.6	10.3 ^a^ ± 0.8	9.8 ^a^ ± 0.5	11.9 ^a^ ± 0.8
Total dry biomass (g)	29.0 ^b^ ± 1.3	37.8 ^a^ ± 1.5	30.8 ^b^ ± 1.7	31.7 ^b^ ± 1.7	37.5 ^a^ ± 2.3
Root/Shoot ratio (g g^−1^)	0.58 ^a^ ± 0.03	0.40 ^b^ ± 0.02	0.51 ^a^ ± 0.03	0.47 ^a^ ± 0.02	0.50 ^a^ ± 0.04

## Supplementary Materials

The following are available online at https://www.mdpi.com/2223-7747/8/3/70/s1. Figure S1, mean foliar nutrient concentration of black spruce seedlings (phosphorus, potassium, calcium) according irrigation × fertilization; Figure S2, number of initial and adventitious roots with cortical cells collapsed or not for seedlings irrigated at 25% water field capacity.
