# Supplementary material for: Influence of Root System Characteristics on Black Spruce Seedling Responses to Limiting Conditions
Source: Plants (Basel). 2019 Mar 19;8(3):70. doi: 10.3390/plants8030070 (PMC6473862; doi:10.3390/plants8030070)
Supplement: Supplementary file 1 [file plants-08-00070-s001.pdf]

Supplementary data

Figure S1. Mean foliar nutrient concentration of black spruce seedlings: (A) P, phosphorus, (B) K, potassium, (C) Ca, calcium according irrigation (25% or 100% water field capacity) × fertilization (F- without, F+ with fertilization). Bars with the same letter indicate a non-significant difference at $P \leq 0.05$.

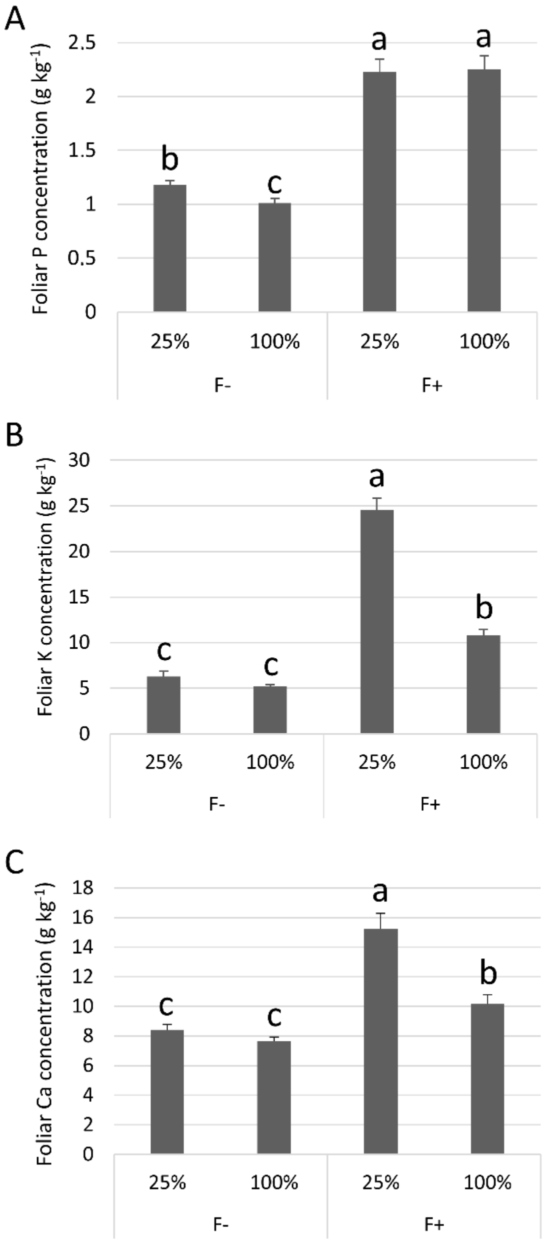

Figure S2. Number of initial and adventitious roots with cortical cells collapsed or not for seedlings irrigated at 25% field capacity. C: containerized seedlings (with a restricted initial root system) and DP: deeply-planted containerized seedlings (with initial and adventitious roots restricted in a root plug).

		**Number of roots with collapsed cortical cells**	**Number of roots with non-collapsed cortical cells**
C seedling	Initial roots	12	6
DP seedling	Initial roots	13	5
	Adventitious roots	18	0
